# Analysis of *CYP2C19* gene polymorphism and influencing factors of pharmacological response of clopidogrel in patients with cerebral infarction in Zhejiang, China

**DOI:** 10.3389/fcvm.2023.1020593

**Published:** 2023-02-02

**Authors:** Yijun Mo, Yao Lu, Fei Guo, Aihua Wu, Yuesong Weng

**Affiliations:** Department of Laboratory Medicine, Ningbo First Hospital, Ningbo Hospital of Zhejiang University, Ningbo, Zhejiang, China

**Keywords:** clopidogrel, *CYP2C19*, logistic regression analysis, homozygous gene, heterozygous gene

## Abstract

**Background:**

Certain genetic and non-genetic factors may cause damaged platelet inhibition by clopidogrel. We aimed to determine the effect of *cytochrome P4502C19* (*CYP2C19*) polymorphism, along with other clinical factors, on the platelet response to clopidogrel in patients with acute ischemic stroke (AIS).

**Methods:**

A total of 214 patients with AIS receiving clopidogrel at a maintenance dose of 75 mg daily admitted to the Ningbo First Hospital between 1 January 2020, and 31 December 2021, were enrolled. Platelet aggregation analysis was performed to determine clopidogrel resistance. Quantitative real-time polymerase chain reaction (QRT-PCR) was used to determine *CYP2C19* genotype. Other laboratory data on complete blood count and biochemical parameters were taken from patient medical files.

**Results:**

Among the 214 AIS patients treated with clopidogrel in the Ningbo population, the incidence of clopidogrel resistance was approximately 43.9%, and the distribution of *CYP2C19* genotypes was highest for CYP2C19(*1/*2) (43.0%), followed by CYP2C19 (*1/*1) (38.8%). The distribution of alleles *1, *2, *3, and *17 was 62.1, 32.5, 4.9, and 0.5%, respectively. A chi-squared test showed that the gene frequencies of alleles *2 and *3 were significantly higher in the clopidogrel-resistant group than in the clopidogrel-sensitive group (*p* < 0.001), and a Mann–Whitney U-test showed that high HCY levels were significantly correlated with clopidogrel resistance (*p* < 0.001). Multi-factor logistic regression analysis demonstrated that mutant heterozygous genotype [OR 2.893; 95% confidence interval (CI) 1.456–5.748; *p* = 0.002], mutant homozygous genotype (OR 4.741; 95% CI 1.828–12.298; *p* = 0.001), and high HCY levels (OR 1.209; 95% CI 1.072–1.362; *p* = 0.002) were significantly associated with clopidogrel resistance.

**Conclusion:**

According to our results, carrying the CYP2C19*2/*3 allele and high HCY levels are independent risk factors for clopidogrel resistance after clopidogrel therapy in patients with AIS. These two factors should be considered prior to clopidogrel administration.

## 1. Introduction

Acute ischemic stroke (AIS) is characterized by a lack of control of the nervous system due to a rapid loss of blood supply to parts of the brain (1). Several studies have shown that AIS can elicit heavy economic and spiritual burdens to affected families and society, and may even lead to death in serious cases (2). In China, there were 2.19 million (95% CI 1.89–2.51) deaths and 45.9 million (39.8–52.3 million) disability adjusted life-years caused by stroke in 2019. In addition, among 28.76 million (25.60–32.21 million) prevalent cases of stroke in 2019, 24.18 million (20.80–27.87 million) were ischemic stroke (IS) (3).

In IS progression, platelet activation plays a critical role and antiplatelet drugs can reduce the risk of stroke by 11–15% (4, 5). Clopidogrel is the most commonly used antiplatelet drug, requiring conversion to its active metabolite, which can bind to the platelet surface of the adenosine diphosphate (ADP) receptor P2Y12, leading to inhibition of platelet aggregation through two sequential oxidative steps (6). This two-stage effect is largely mediated by the metabolizing enzyme *CYP2C19*. Cytochrome P450 comprises a family of enzymes that are responsible for most drug metabolism reactions occurring in humans. Although many cytochrome P450 isoforms exist, most reactions are carried out by *CYP3A4, CYP2C19, CYP2C9*, and *CYP2D6* (7).

*CYP2C19* is the most potent genetic determinant of the clopidogrel response (8). Alleles are classified into the following functional groups based on published *in vitro* and/or *in vivo* data: normal function (e.g., CYP2C19*1), which encodes a functional *CYP2C19* enzyme that converts the clopidogrel prodrug into its active metabolite; increased function (e.g., CYP2C19*17), resulting in a protein with increased enzymatic activity; and no function (e.g., CYP2C19*2 and *3), which is associated with decreased enzymatic activity (8, 9). This implies that *2/*3 allele carriers are *CYP2C19* loss-of-function (LOF) alleles. It has been demonstrated that *CYP2C19* LOF allele carriers have an increased risk of composite vascular events and new stroke in IS patients treated with clopidogrel when compared to non-carriers (10).

Clopidogrel resistance (CR) refers to patients who continue to present with a thrombotic event after clopidogrel treatment, and laboratory tests have shown that platelet function is not inhibited (11). This non-responsiveness to clopidogrel may be multifactorial, and several genetic and non-genetic determinants may contribute to this effect (12). To investigate this, we studied 214 patients with AIS at the Ningbo First Hospital, Ningbo, Zhejiang, China. We aimed to determine the frequency of genetic polymorphisms of *CYP2C19* in Zhejiang’s population presenting with IS and to assess the contribution of this polymorphism, along with other clinical factors (high density lipoproteins, low density lipoproteins, homocysteine, triglycerides, total cholesterol, neutrophils/lymphocytes, platelet count, HbA1c, glycosylated hemoglobin) of the clopidogrel response. The results of this study will help improve individualized antiplatelet therapy regimens for patients with IS and reduce adverse side effects.

## 2. Materials and methods

### 2.1. Materials

The following materials were utilized in our study: MX3000P PCR amplifier (Agilent Stratagene, Santa Clara, CA, USA); AG800 automatic platelet aggregation instrument (Techlink med, China); CYP2C19 Gene Test Kit (Yzy med, China); and Platelet aggregation function assay (ADP-activated turbidimetric assay kit, Techlink med China).

### 2.2. Study population

We included 214 patients with AIS admitted to Ningbo First Hospital from 1 January 2020, to 31 December 2021. Studies have shown that CR is associated with the dose used and increasing the maintenance doses of clopidogrel in patients who carry a CYP2C19*2 allele will correspondingly reduce platelet reactivity (13). The recruited patients were taking clopidogrel at a standard maintenance dose of 75 mg daily. Based on their platelet aggregation test results, the patients were divided into two groups: the clopidogrel-resistant group and the clopidogrel-sensitive group.

We included patients as follows: those who were taking clopidogrel 75 mg/day for more than seven days; and those who had suffered an acute cerebral infarction confirmed by cranial CT or MRI. we excluded those who had taken glycoprotein IIb/IIIa receptor antagonists or other thienopyridines within the last week; those with abnormal liver, kidney, spleen, and hematopoietic function; patients with a platelet count more than 500 × 10^9^/L or less than 100 × 10^9^/L; patients with malignant tumor, history of hemorrhage, or intracranial hemorrhage within the last three months; and those with atrial fibrillation.

### 2.3. Data collection

This study was approved by the Ethics Committee of Ningbo First Hospital (Approval No. 2022RS009). Written informed consent was obtained from all patients. Data were collected from the medical files of patients and, in addition to complete blood counts and biochemical parameters [high density lipoprotein (HDL), low density lipoprotein (LDL), homocysteine (HCY), triglycerides (TGs), total cholesterol (TC), HbA1c, and glycosylated hemoglobin], data on gender, age, clopidogrel use, medical history, and surgical history were collected.

### 2.4. Quantitative real-time polymerase chain reaction for the CYP2C19 gene

DNA was extracted from whole blood samples using a TIANamp Blood DNA kit (TIANGEN, Beijing, China). Genotyping was performed using the Human CYP2C19 Gene Test Kit (YZYMED, Wuhan, China). The final reaction volume of 25 μl consisted of 2 μl of template DNA and 23 μl PCR reaction mix designed for three SNPs. The amplification reaction was carried out as follows: an initial denaturation at 95°C for 10 min, followed by 40 cycles of denaturation at 95°C for 15 s, and annealing/extension at 60°C for 1 min.

### 2.5. Determination criteria

Platelet aggregation >50% is considered as CR (14). According to standard protocols (15), platelet aggregation was assessed using light transmission aggregometry (LTA) which measures platelet coaggregation in platelet-rich plasma in response to ADP.

### 2.6. Statistical analyses

SPSS 22.0 statistical software was used, and missing values were replaced by the mean value method. The count data were expressed as percentages and cases, and the χ^2^ test was used for comparison between groups. The measurement data were expressed as “x ± s,” and the *t*-test and Mann–Whitney U-test was used for comparison between groups. Logistic multi-factor analysis was used, and the Hardy–Weinberg law of genetic equilibrium was applied to test whether the study subjects were representative of the group.

## 3. Results

Among the 214 study patients, 156 were male and 58 were female; ages ranged from 18 to 89 years, with a mean age of 63.16 ± 11.32 years. In addition, of these patients, 147 had hypertension (68.7%) and 46 were diabetes (21.5%). A comparison of basic data between the clopidogrel reaction group and clopidogrel-resistant group is shown in Table 1.

**TABLE 1:** Comparison of basic data between the clopidogrel reaction group and clopidogrel resistance group.

Basic data	Clopidogrel-resistant (*n* = 94)	Clopidogrel-sensitive (*n* = 120)
Age	63.14 ± 10.710	63.39 ± 11.822
Male (*n*)	69	87
Female (*n*)	25	33
Diabetes [*n* (%)]	26 (27.6%)	20 (20.0%)
Hypertension [*n* (%)]	61 (64.8%)	86 (71.6%)

The distribution of the *CYP2C19* gene polymorphism in patients is shown in Table 2. We found that, in the 214 patients, 83 (38.8%) carried wild-type genes, 100 cases (46.7%) carried mutant heterozygous genes, and 31 cases (14.5%) carried mutant homozygous genes. Among the 100 patients carrying mutant heterozygous genes, 92 carried (*1/*2), and eight carried (*1/*3). Among the 33 patients carrying mutant homozygous genes, 17 carried (*2/*2), 12 patients carried (*2/*3), one patient carried (*2/*17), and one patient carried (*3/*17). Furthermore, according to the *CYP2C19* Diplotype-Phenotype online Table (16, 17), we divided the genetic phenotypes into three categories as normal metabolizers (NM), intermediate metabolizers (IM), and poor metabolizers (PM). We also calculated genotype frequencies as follows: the CYP2C19*1 allelic frequency was 62.1%; the CYP2C19*2 allelic frequency was 32.5%; the CYP2C19*3 allelic frequency was 4.9%; and the CYP2C19*17 allelic frequency was 0.5%.

**TABLE 2:** Genotypic and allelic frequencies and phenotypes of IS patients.

Genotype	Distribution, *n* (%)	Phenotype	Allele	Frequency, %
CYP2C19(*1/*1)	83(38.8)	NM	*1	62.1
CYP2C19(*1/*2)	92(43.0)	IM	*2	32.5
CYP2C19(*1/*3)	8(3.7)	IM	*3	4.9
CYP2C19(*2/*2)	17(7.9)	PM	*17	0.5
CYP2C19(*2/*3)	12(5.6)	PM		
CYP2C19(*2/*17)	1(0.5)	IM		
CYP2C19(*3/*17)	1(0.5)	IM		

NM, normal metabolizers; IM, intermediate metabolizers; PM, poor metabolizers.

Since the *17 allele is associated with increased activation of clopidogrel, and whether its increased function can compensate for the LOF alleles remains unknown (18), the statistical results in Table 3 did not include patients carrying the CYP2C19*17 allele. In Table 3, among the 93 clopidogrel-resistant patients, 19 (20.4%) were wild-type gene carriers, 55 (59.2%) were mutant heterozygous genes carriers, and 19 (20.4%) were mutant homozygous genes carriers. Among the 119 clopidogrel-sensitive cases, 64 (53.8%) were wild-type gene carriers; 45 (37.8%), mutant heterozygous gene carriers; and 10 (8.4%), mutant homozygous gene carriers. There were significant differences in genotype and allele frequency distribution between the clopidogrel-sensitive and clopidogrel-resistant groups (*p* < 0.05). Heterozygote carriers were more resistant to clopidogrel than wild-type carriers (*p* < 0.001), and *2/*3 allele carriers were more resistant to clopidogrel than *1 allele carriers (*p* < 0.001).

**TABLE 3:** Genotypes distribution and allele frequencies of *CYP2C19* gene polymorphism.

Genotype and alleles	Clopidogrel-resistant (*n* = 93)	Clopidogrel-sensitive (*n* = 119)	x^2^	*P*-value
wt	19 (20.4%)	64 (53.8%)		
htr	55 (59.2%)	45 (37.8%)	25.38	<0.001
homo	19 (20.4%)	10 (8.4%)		
*1	93 (50.0%)	173 (72.7%)	22.99	<0.001
*2,*3	93 (50.0%)	65 (27.3%)		

Wt, wild-type genes (CYP2C19(*1/*1)); htr, mutant heterozygous genes (CYP2C19(*1/*2), CYP2C19(*1/*3)); homo, mutant homozygous genes (CYP2C19(*2/*2), CYP2C19(*2/*3)).

We also compared platelet count, TC, TG, HDL, LDL, GLU, HbA1c, and HCY levels in the clopidogrel-resistant and clopidogrel-sensitive groups in Table 4, and the statistical results showed that only HCY levels were significantly correlated with CR. This was significantly higher in the clopidogrel-resistant group than in the clopidogrel-responsive group, with a statistically significant difference (Z = −4.588, *p* < 0.001).

**TABLE 4:** Comparison of laboratory data between the clopidogrel-resistant and -sensitive groups.

Lab variable	Clopidogrel-resistant (*n* = 94)	Clopidogrel-sensitive (*n* = 120)	T/Z	*p*
TC (mmol/L, x ± s)	4.3986 ± 1.23605	4.1404 ± 1.16215	T = 1.569	0.118
TG (mmol/L, x ± s)	1.4289 ± 0.70533	1.5135 ± 0.81398	T = −0.799	0.425
Platelets count (× 10^9^, x ± s)	232.89 ± 60.318	228.29 ± 65.578	T = 0.528	0.598
HDL (mmol/L, x ± s)	1.0360 ± 0.23424	1.0293 ± 0.23529	Z = −0.046	0.964
LDL (mmol/L, x ± s)	2.8825 ± 0.86941	2.7707 ± 0.85300	Z = −0.952	0.341
GLU (mmol/L, x ± s)	5.788 ± 1.7893	5.765 ± 1.6661	Z = −0.448	0.654
HbA1C (mmol/L, x ± s)	6.518 ± 1.5136	6.283 ± 1.3284	Z = −0.795	0.427
HCY (μmol/L, x ± s)	11.8669 ± 4.7168	9.8293 ± 2.5016	Z = −4.588	0.000

TC, total cholesterol; TG, triglycerides; HDL, high density lipoprotein; LDL, low density lipoprotein; GLU, glucose; HbA1c, glycosylated hemoglobin; HCY, homocysteine.

Binary logistic regression analysis was performed for age, gender, diabetes, hypertension, genotype, HDL, LDL, HCY, TG, TC, N/L, MPV/PLT, and HbA1c as independent variables. As for Table 3, we did not analyze patients with the *17 allele. As shown in Table 5, several risk factors, such as mutant heterozygous, mutant homozygous, and high HCY levels, were significantly correlated with CR (*p* < 0.05): mutant heterozygous genotype (OR 2.893; 95% CI 1.456–5.748; *p* = 0.002), mutant homozygous gene (OR 4.741; 95% CI 1.828–12.298; *p* = 0.001), and high HCY levels (OR 1.209; 95% CI 1.072–1.362; *p* = 0.002).

**TABLE 5:** Analysis of factors associated with clopidogrel resistance.

Factor	Univariate		Multi-univariate	
	***P*-value**	**OR (95% CI)**	***P*-value**	**OR (95% CI)**
Gender	0.999	1.000 (0.542–1.843)		
Age	0.555	0.849 (0.493–1.462)		
Diabetes II	1.194	1.535 (0.804–2.927)		
Hypertension	0.283	0.727 (0.406–1.301)		
htr	0.000	4.117 (2.158–7.855)	0.002	2.893 (1.456–5.748)
homo	0.000	6.400 (2.548–16.078)	0.001	4.741 (1.828–12.298)
HDL	0.959	1.031 (0.321–3.311)		
LDL	0.455	1.132 (0.818–1.567)		
HCY	0.000	1.295 (1.153–1.455)	0.002	1.209 (1.072–1.362)
TG	0.390	0.853 (0.583–1.226)		
TC	0.159	1.183 (0.937–1.493)		
N/L	0.555	0.964 (0.855–1.088)		
PLT	0.516	1.001 (0.997–1.006)		
HbA1C	0.220	1.128 (0.930–1.369)		

htr, mutant heterozygous genes (CYP2C19(*1/*2), CYP2C19(*1/*3)); homo, mutant homozygous genes (CYP2C19(*2/*2), CYP2C19(*2/*3)); HDL, high density lipoprotein; LDL, low density lipoprotein; HCY, homocysteine; TG, triglycerides; TC, total cholesterol; N/L, neutrophils/lymphocyte; PLT, platelets count; HbA1c, glycosylated hemoglobin.

## 4. Discussion

Among the 214 AIS patients treated with clopidogrel in the Ningbo population, the incidence of CR was approximately 43.9%, and the distribution of *CYP2C19* genotypes was highest for CYP2C19(*1/*2). The distribution of alleles *1, *2, *3, and *17 was 62.1%, 32.5%, 4.9%, and 0.5%, respectively. The gene frequencies of alleles *2 and *3 were significantly higher in the clopidogrel-resistant group than in the clopidogrel-sensitive group, and high HCY levels were significantly correlated with CR. Mutant heterozygous genotype, mutant homozygous genotype, and high HCY levels were significantly associated with CR.

In 2019, worldwide stroke remained the second-leading cause of death. Moreover, IS remained the largest proportion of all new strokes (62.4% of all stroke incidents in 2019) (19). Platelets are activated by ADP, collagen, and the arachidonic acid metabolite thromboxane, A2. Activated platelets aggregate to form blood clots, leading to AIS (20). Clopidogrel is commonly used as an antiplatelet agent and its active metabolite can specifically and irreversibly block the binding of ADP to platelet receptors, thereby inhibiting platelet aggregation and reducing the risk of AIS (21). Although the antiplatelet mechanism of clopidogrel is well established, observational studies have shown that there is considerable variability in response to clopidogrel after antiplatelet treatment, and a suboptimal response may lead to recurrent ischemic events in cardiovascular disease (22).

From our results, wild-type *1 had the highest proportion of reported rates, similar to those previously published (23). Using LTA, a 57.3% incidence of *CYP2C19* LOF allele carriers were resistant to clopidogrel. Our results also showed that CYP2C19*2 or CYP2C19*3 allele carriers were significantly more resistant to clopidogrel than *1 allele carriers (*p* < 0.05). Furthermore, logistic multi-factor analysis also demonstrated that *2 or *3 allele carriers were significantly correlated with CR (*p* < 0.05), indicating that CYP2C19*2 or CYP2C19*3 allele carriers were independent risk factors for CR. It is worth mentioning that CYP2C19 (*3/*17) genotype carrier was resistant to clopidogrel, but CYP2C19 (*2/*17) carrier was sensitive to clopidogrel in our study. Limited data indicates that increased function (e.g., CYP2C19*17) may not compensate for *CYP2C19* LOF alleles (18).

In addition to the genetic polymorphism of the drug metabolizing enzyme CYP2C19, many other factors have been reported to be related to clopidogrel response. Several studies have shown that diabetes mellitus (DM) is associated not only with an increased platelet reactivity but also with a decreased responsiveness to clopidogrel in AIS patients taking dual antiplatelet therapy (DAPT) (24). In platelets from Type 2 DM patients, the P2Y12 pathway appears to be upregulated and less sensitive to P2Y12 inhibition (25). Moreover, hyperglycemic states induce glycation of platelet GPs, which changes platelet structure and weakens the antiplatelet aggregation effect of clopidogrel (26). However, our results demonstrate that Type 2 diabetes, high HbA1c levels, and high GLU levels had no correlation with CR. Another study found that systemic inflammation and insulin resistance play an important role in the resistance of diabetic patients to clopidogrel (27). However, Ang et al. (28) reported that inflammatory markers, such as platelet counts and white blood cells, have no relation to CR, which is consistent with our study.

A previous study found that patients with minor stroke/transient ischemic attack with overweight/obesity did not benefit from clopidogrel therapy, and that body mass index (BMI) was a major independent predictor of an insufficient antiplatelet response to clopidogrel (29). Higher BMI was directly related to hypercholesterol and TGs, and inversely related to HDL cholesterol (30). Therefore, we tested these biochemical parameters to find a correlation between the response to clopidogrel and these parameters. However, among the tested biochemical parameters (TC, TG, HDL, and LDL), none were found to be associated with a decrease in clopidogrel response.

Hypertension is a risk factor for recurrent stroke (31), and researchers have found that CR is associated with hypertension. Kim et al. (32) suggested that high adhesivity and aggregability of platelets in hypertensive patients may account for clopidogrel non-responsiveness. Another study also believed that hypertension caused increased shear stress in blood vessels, which could reactivate platelets and cause CR (33). However, our study found no significant association between the response to clopidogrel and hypertension.

Plasma HCY is an established risk factor for cardiovascular disease (CVD) and stroke (34). HCY is a non-essential amino acid; however, it does not participate in protein synthesis. Studies have demonstrated that high HCY level can induce vascular injuries leading to intimal thickening, rupture of the elastic layer, smooth muscle hypertrophy, significant platelet dysfunction, and formation of platelet-rich occlusive thrombi (35, 36). Elevated HCY could promote oxygen free radical generation and damage the vascular endothelium leading to platelet aggregation and thrombosis (37). Furthermore, a meta-analysis indicated that a non-linear association could exist between HCY levels and risk of IS (38). Therefore, we analyzed the correlation between HCY levels and CR, and confirmed that high HCY levels significantly correlated to CR (OR 1.209; 95% CI 1.072–1.362; *p* = 0.002), which was consistent with another study (39). An experimental study revealed that high HCY levels cause oxidative stress, platelet activation, endothelium dysfunction, chronic inflammation, and, therefore, impaired platelet inhibition (40).

Our study had some limitations. First, this was a single center, retrospective study, and the subjects were mainly derived from the local Han population, so extending these results to other cohorts may require careful interpretation and further study. Second, confounding factors such as liver function, the use of proton pump inhibitors, and other genetic factors might influence the responsiveness to clopidogrel (8). Third, the sample size was relatively small. In particular, the proportion of women was low. Lastly, LTA is defined as the gold standard method in platelet-rich plasma in response to ADP, but this test is poorly standardized with short sample stability and is affected by numerous pre-analytical variables (41). Therefore, there are no exact definitions of CR.

In conclusion, carrying the *CYP2C19* LOF allele and high HCY levels are independent risk factors for CR in patients with AIS. Additionally, the *CYP2C19* genotype (OR = 4.741) has a more dominant influence than HCY levels (OR = 1.209), although the relationship between platelet inhibition rates and actual clinical events has not been established, and the factors contributing to CR have not been identified. Based on the purpose of precision therapy, clinical guidance is required to adjust treatment strategies according to these laboratory detection indicators.

## Data availability statement

The original contributions presented in this study are included in the article/Supplementary material, further inquiries can be directed to the corresponding author.

## Ethics statement

The studies involving human participants were reviewed and approved by Ethics Committee of Ningbo First Hospital affiliation: Ningbo First Hospital, Ningbo Hospital of Zhejiang University. Written informed consent for participation was not required for this study in accordance with the national legislation and the institutional requirements.

## Author contributions

YM conceived and designed the experiments. FG conducted the experiments. AW and YW analyzed the data. YL wrote the first draft of the manuscript. All authors reviewed and edited the manuscript and approved the final version.
